# GCase and LIMP2 Abnormalities in the Liver of Niemann Pick Type C Mice

**DOI:** 10.3390/ijms22052532

**Published:** 2021-03-03

**Authors:** Martijn J. C. van der Lienden, Jan Aten, André R. A. Marques, Ingeborg S. E. Waas, Per W. B. Larsen, Nike Claessen, Nicole N. van der Wel, Roelof Ottenhoff, Marco van Eijk, Johannes M. F. G. Aerts

**Affiliations:** 1 Department Medical Biochemistry, Leiden University, 2333 CC Leiden, The Netherlands; m.j.c.van.der.lienden@lic.leidenuniv.nl (M.J.C.v.d.L.); m.c.van.eijk@LIC.leidenuniv.nl (M.v.E.); 2 Department of Pathology, Amsterdam UMC, University of Amsterdam, 1100 DD Amsterdam, The Netherlands; j.aten@amsterdamumc.nl (J.A.); i.s.e.waas@amsterdamumc.nl (I.S.E.W.); p.w.larsen@amsterdamumc.nl (P.W.B.L.); n.claessen@amsterdamumc.nl (N.C.); 3 Chronic Diseases Research Centre, Universidade NOVA de Lisboa, 1150-082 Lisbon, Portugal; andre.marques@nms.unl.pt; 4 Electron Microscopy Center Amsterdam, Department of Medical Biology, Amsterdam UMC, 1100 DD Amsterdam, The Netherlands; n.n.vanderwel@amsterdamumc.nl; 5 Department of Medical Biochemistry, Amsterdam UMC, University of Amsterdam, 1100 DD Amsterdam, The Netherlands; r.ottenhoff@amc.uva.nl

**Keywords:** lysosome, NPC, LIMP2, storage, macrophage, NPC1, GPNMB, GCase

## Abstract

The lysosomal storage disease Niemann–Pick type C (NPC) is caused by impaired cholesterol efflux from lysosomes, which is accompanied by secondary lysosomal accumulation of sphingomyelin and glucosylceramide (GlcCer). Similar to Gaucher disease (GD), patients deficient in glucocerebrosidase (GCase) degrading GlcCer, NPC patients show an elevated glucosylsphingosine and glucosylated cholesterol. In livers of mice lacking the lysosomal cholesterol efflux transporter NPC1, we investigated the expression of established biomarkers of lipid-laden macrophages of GD patients, their GCase status, and content on the cytosol facing glucosylceramidase GBA2 and lysosomal integral membrane protein type B (LIMP2), a transporter of newly formed GCase to lysosomes. Livers of 80-week-old *Npc1*^−/−^ mice showed a partially reduced GCase protein and enzymatic activity. In contrast, GBA2 levels tended to be reciprocally increased with the GCase deficiency. In *Npc1*^−/−^ liver, increased expression of lysosomal enzymes (cathepsin D, acid ceramidase) was observed as well as increased markers of lipid-stressed macrophages (GPNMB and galectin-3). Immunohistochemistry showed that the latter markers are expressed by lipid laden Kupffer cells. Earlier reported increase of LIMP2 in *Npc1*^−/−^ liver was confirmed. Unexpectedly, immunohistochemistry showed that LIMP2 is particularly overexpressed in the hepatocytes of the *Npc1*^−/−^ liver. LIMP2 in these hepatocytes seems not to only localize to (endo)lysosomes. The recent recognition that LIMP2 harbors a cholesterol channel prompts the speculation that LIMP2 in *Npc1*^−/−^ hepatocytes might mediate export of cholesterol into the bile and thus protects the hepatocytes.

## 1. Introduction

The liver plays a key role in bodily cholesterol homeostasis. It produces, metabolizes, secretes, and endocytoses cholesterol. Following the endocytic uptake by hepatocytes and Kupffer cells, cholesterol is exported from the endo(lysosomes) to the cytosol. This process is carried out by the lysosomal proteins NPC1 and NPC2 (NPC intracellular cholesterol transporter 1 and 2) [1]. The latter protein transfers cholesterol from (endo)lysosomal luminal membrane vesicles to the lysosomal membrane protein NPC1 that subsequently mediates its efflux from the lysosome. Deficiencies in either NPC1 or NPC2 cause Niemann–Pick disease type C (NPC), a lysosomal storage disorder characterized by accumulation of cholesterol that is accompanied by increases in other lipids including sphingomyelin and glucosylceramide (GlcCer) [2]. As compared to Kupffer cells, NPC1-deficient hepatocytes demonstrate less prominent lysosomal storage [3]. The secondary accumulation of GlcCer in NPC liver suggests interaction between cholesterol and GlcCer metabolism. Several other findings point to this. For example, the activity of glucocerebrosidase (GCase), the lysosomal β-glucosidase degrading GlcCer to ceramide, is found to be reduced in NPC [4,5,6]. Indeed, like GCase-deficient Gaucher disease (GD) patients, NPC patients manifest with elevated glucosylsphingosine (GlcSph) [7]. GlcSph is also increased in tissues and plasma of NPC mice. GlcSph is formed by acid ceramidase from GlcCer accumulating in lysosomes [8]. GCase is known to also act as transglucosidase, generating glucosylated cholesterol (GlcChol) during lysosomal cholesterol accumulation [9]. Consequently, GlcChol is elevated more than thirty-fold in livers of NPC mice and significantly increased in plasma of NPC mice and patients [9]. Under normal conditions GlcChol is synthesized by the cytosol-facing enzyme GBA2 (glucosylceramidase beta 2) and degraded to glucose and cholesterol by GCase in lysosomes [10].

The enzyme GCase is bound to the membrane protein LIMP2 (lysosomal integral membrane protein type B, encoded by the SCARB2 gene) during its transport to the lysosome [11]. Following folding in the endoplasmic reticulum, GCase associates to LIMP2 and the complex is routed to lysosomes where dissociation is favored by the local low pH [12,13]. Recently it has become apparent that LIMP2 contains a putative channel allowing transport of cholesterol molecules [14]. During deficiency of NPC1, LIMP2 appears to be involved in cholesterol efflux from lysosomes [14]. This is substantiated by the observation that a dual deficiency in NPC1 and LIMP2 results in a more prominent SREBP2 (sterol regulatory element binding protein 2)-driven induction of HMG-CoA reductase transcription, a classic readout for impaired cholesterol efflux from lysosomes [14]. Of note, LIMP2-deficient mice show no marked abnormalities in cholesterol homeostasis, suggesting that NPC1 is normally sufficient to govern cholesterol efflux from lysosomes [9]. Of interest, recent proteomics studies by Cologna in NPC1 mice have revealed an increased level of LIMP2 in liver as well as in cerebral cortex and cerebellum [15,16].

A striking similarity between NPC and GCase-deficiencies (GD) is the overexpression of specific proteins by lipid-laden macrophages. In Gaucher disease, GlcCer-laden macrophages excessively produce the chitinase chitotriosidase, the chemokine CCL18 (C−C motif chemokine ligand 18) and GPNMB (glycoprotein nmb) [17,18,19,20,21]. In plasma of symptomatic Gaucher patients, chitotriosidase, CCL18, and a soluble fragment of GPNMB are spectacularly increased and these abnormalities are exploited as biomarkers [22]. Increased plasma levels of these biomarkers also occur in NPC [23,24]. Finally, increased metabolism of GlcCer by the cytosol-facing glucosylceramidase GBA2, which is observed during GCase deficiency, has also been noted in brains of NPC mice [10]. Pharmacological inhibition of GBA2 activity with a hydrophobic iminosugar [25] or GBA2 gene ablation remarkably ameliorates neuropathology in NPC mice and increases their life span [10].

In the present study, we examined the status of GCase in the liver of mice lacking the NPC1 protein and determined the expression of biomarkers of lipid-laden macrophages. Furthermore, we investigated the status of GBA2 and LIMP2. Our study revealed an increased expression of biomarkers of lipid-stressed macrophages, but it also disclosed remarkable upregulation of LIMP2 in hepatocytes.

## 2. Results

### 2.1. Structural Analysis of Npc1^−/−^ Liver

We earlier described the development of abnormalities in glycosphingolipids in the livers of *Npc1^−/−^* mice, coinciding with cholesterol accumulation [24]. At an age of 12 weeks, the liver GlyCer was 606.4 (mean) ± 152.3 (SD) nmol/g wet weight, being 14.9 higher than in corresponding livers of wild type BALB/c mice. Liver GlySph, deacylated GlyCer formed by acid ceramidase in lysosomes [7,8], was 3121.2 ± 453.3 pmol/g wet weight, being 206.3-fold elevated compared to normal. Finally, liver glycosylated cholesterol [9] was 25.4 ± 6.4 nmol/g wet weight, being 32-fold elevated compared to normal.

**Figure 1:**
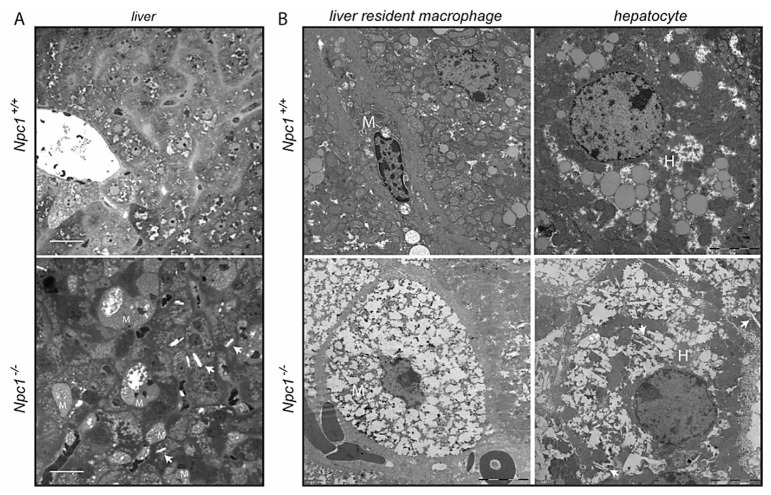
Structural analysis of liver of 80-day-old *Npc1^+/+^* and *Npc1^−/−^* mice; (**A**) micrographs of toluidine blue-stained sections. Scale bar = 25 µm; (**B**) transmission electron microscopy (TEM) micrographs of liver resident macrophages (M) and hepatocytes (H). Scale bar = 5 µm. Arrows indicate clefts in hepatocytes of *Npc1^−/−^* liver.

At the age of 70–80 days the pathology of the liver of *Npc1^−/−^* was prominent and the animals did not suffer from weight loss yet. The livers of 80-day-old *Npc1^−/−^* mice and corresponding wt mice were analyzed in more detail. The *Npc1^−/−^* livers showed clusters of enlarged macrophages as visualized by toluidine blue staining Figure 1A. Ultrastructural analysis of the tissue by transmission electron microscopy (TEM) confirmed the presence of characteristic storage cells of macrophage origin with vacuoles that are based on the ultrastructure of the nucleus in *Npc1^−/−^* liver (Figure 1B). A markedly altered ultrastructure was also observed with respect to *Npc1^−/−^* hepatocytes (Figure 1B). Of note, the *Npc1^−/−^* hepatocytes contained clefts/electron-lucent vacuoles suggestive of deposition of cholesterol crystals (Figure 1A,B) [26,27,28].

### 2.2. Lysosomal GCase and Cytosol Facing GBA2 in NPC Liver

**Figure 2:**
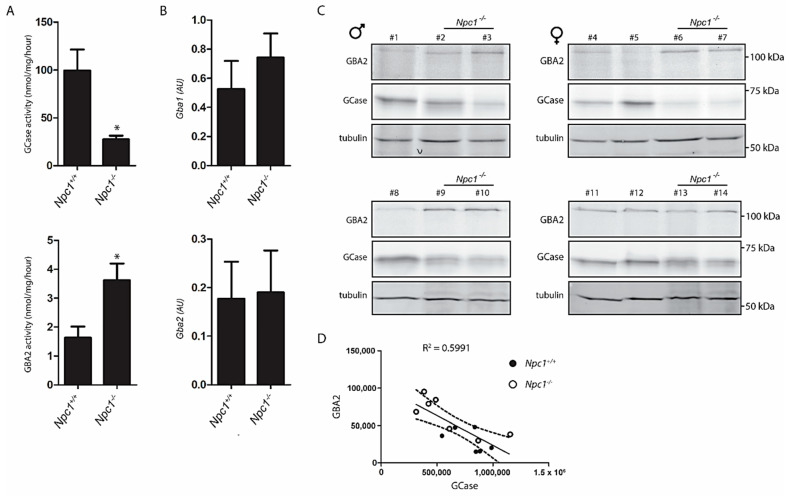
Lysosomal GCase and cytosol facing GBA2 in *Npc1^+/+^* and *Npc1^−/−^* mouse liver; (**A**) GCase activity was measured in liver lysates of *Npc1^+/+^* and *Npc1^−/−^* mice with 4MU-β-Glc substrate as described in the Material and Methods section; (**B**) real-time (Rt) qPCR mRNA analysis of Gba1 and Gba2 gene expression in lysates of the same livers. Values represent relative numbers compared to ribosomal gene Rplp0; (**C**) GCase and GBA2 in aliquots of the same lysates were labelled with GCase and GBA2 specific activity-based probe (ABP) and subsequently visualized by fluorescence scanning after SDS-PAGE. # *Npc1^+/+^*: 1, 4, 5, 8, 11, 12; # *Npc1^−/−^*: 2, 3, 6, 7, 9, 10, 13, 14; (**D**) reciprocal correlation between overall active GCase and GBA2 molecules in liver lysates of *Npc1^+/+^* and *Npc1^−/−^* mice based on quantification of labelled bands in (**C**). AU: arbitrary units. Significance (*p ≤* 0.05) is indicated by astesisk.

The enzymatic activity of GCase in total *Npc1*^−/−^ liver extract was on average 60% reduced (Figure 2A). This reduction is similar to earlier observations made with *Npc1*^−/−^ brain [10]. Of note, the expression of the *Gba1* gene tended to be upregulated in the *Npc1*^−/−^ liver (Figure 2B), which indicates that GCase in the *Npc1*^−/−^ liver is post-transcriptionally reduced.

An activity-based probe (ABP), which covalently binds to the catalytic nucleophile of retaining β-glucosidases, was used to simultaneously visualize active GCase (59−66 kDa) and GBA2 (110 kDa) enzyme molecules in extracts of the various livers. Although considerable interindividual variation was noted for levels of GCase and GBA2 activities in livers (Figure 2C, quantified in Supplementary Figure S1), a reduction in GCase was found to correlate with an elevation of GBA2 (Figure 2D). Of note, while enzyme activity measurement and ABP labelling reveal a reduction of active GCase in the *Npc1*^−/−^ liver, the *Gba1* mRNA determined by qPCR did not, suggesting that the decrease of GCase activity is not due to reduced biosynthesis but more likely to a reduced stability of the enzyme.

### 2.3. Response to Lysosomal Storage in NPC Liver

**Figure 3:**
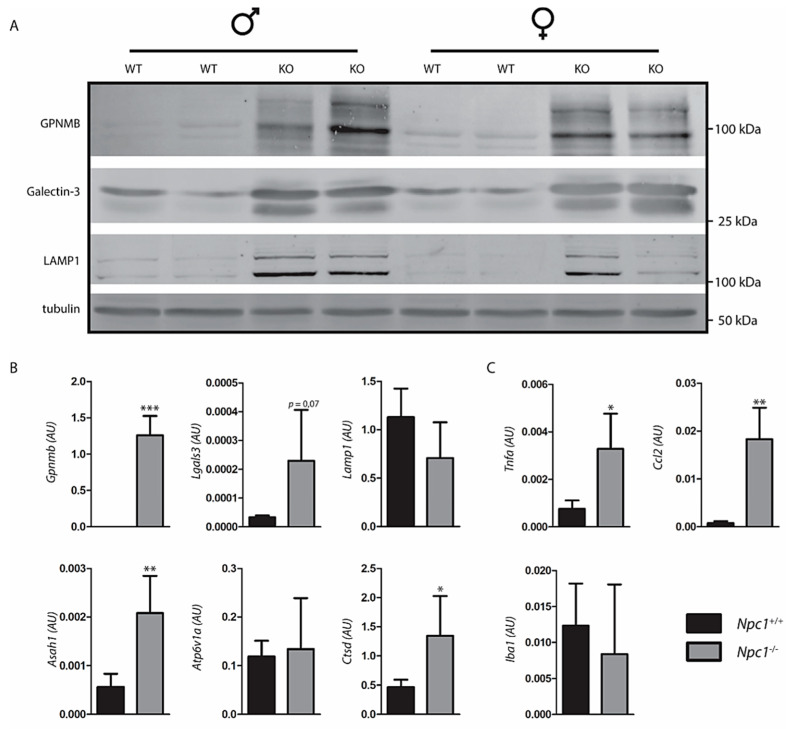
Response to lysosomal storage in Niemann–Pick type C (NPC) liver; (**A**) Western blot analysis of lysosome storage associated proteins in lysates of *Npc1^+/+^* and *Npc1^−/−^* mouse liver; (**B**) real-time (Rt) qPCR RNA analysis of the same livers regarding lysosomal storage associated genes; (**C**) inflammatory genes. Values represent relative numbers compared to ribosomal gene Rplp0. AU: arbitrary units; Significance is indicated by asterisks, * *p ≤* 0.05, ** *p ≤* 0.01, *** *p ≤* 0.001.

*Npc1*^−/−^ mice exhibit a striking degree of storage in macrophages. Lysosome perturbation by storage is usually accompanied by either increased lysosomal swelling, biogenesis or both, and expression of particular markers such as GPNMB [24,29]. Employing Western blotting, a clear increase in GPNMB in *Npc1*^−/−^ livers was demonstrable (Figure 3A). Likewise, the lysosomal membrane protein LAMP1 (lysosomal associated membrane protein 1) and galectin-3, a protein associated with lysosome stress, were found to be increased as visualized by Western blotting. Next, the expression of illustrative genes encoding lysosomal proteins was analyzed (Figure 3B). We noted markedly increased expression of the *Gpnmb* and *Lgals3* genes, coding for GPNMB and galectin-3. No significant upregulation of expression of the genes *Lamp1*, encoding the lysosomal membrane protein LAMP1 and *Atp6v1a*, encoding the vATPase subunit H, was detected. However, an increased expression of *Asah1*, encoding acid ceramidase, and *Ctsd*, encoding cathepsin D was observed in the *Npc1*^−/−^ mouse livers. Furthermore, the *Npc1*^−/−^ mouse livers showed increased expression of genes coding for proteins implicated in inflammation TNFα, CCL2 (Figure 3C). No clear changes were observed in expression of the *Iba1* gene, which encodes a commonly used marker of macrophages (Figure 3C).

### 2.4. LIMP2 Upregulation in NPC Liver but Not in Cultured Cells with Pharmacologically Induced Lysosomal Cholesterol Accumulation

**Figure 4:**
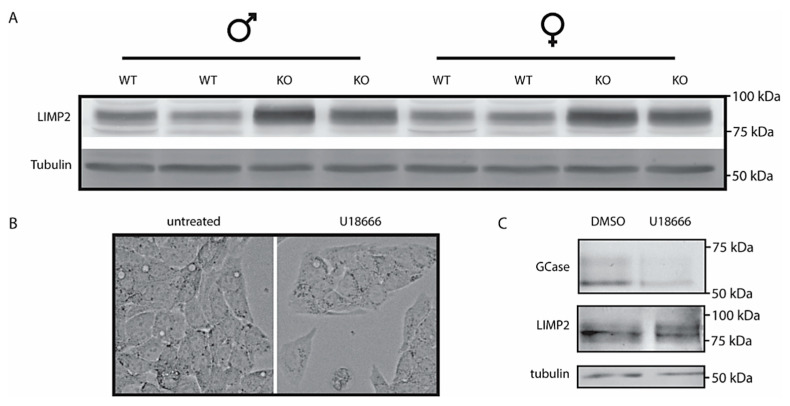
Lysosomal integral membrane protein type B (LIMP2) in *Npc1^−/−^* liver and U18666A treated HEPG2 cells; (**A**) Western blot analysis of LIMP2 in lysates of *Npc1^+/+^* and *Npc1^−/−^* mouse liver; (**B**) phase contrast microscopy pictures of HEPG2 cells treated with NPC1-inhibitor U18666A (magnification of 63×); (**C**) GCase in HEPG2 lysates was labelled with GCase-specific ABP and subsequently visualized by fluorescence scanning after SDS-PAGE. LIMP2 was analyzed by Western blotting of the same wet gel slab as described in the Materials and Methods section.

The analysis of LIMP2 by Western blotting revealed a clear increase in the membrane protein in the *Npc1*^−/−^ livers (Figure 4A, quantified in Supplementary Figure S2). To further study this unexpected finding, we analyzed cultured cells exposed to U18666A, an agent causing lysosomal cholesterol accumulation [9]. No major change in morphology was detected in HEPG2 cells treated with U18666A (Figure 4B). Treatment of HepG2 cells with U18666A caused a reduction in GCase as detected with activity-probe labelling but no change in LIMP2 as detected by Western blotting (Figure 4C). In other words, the findings with NPC1-deficient liver for LIMP2 were not fully recapitulated.

### 2.5. Immunohistochemical Analysis of NPC Liver

**Figure 5:**
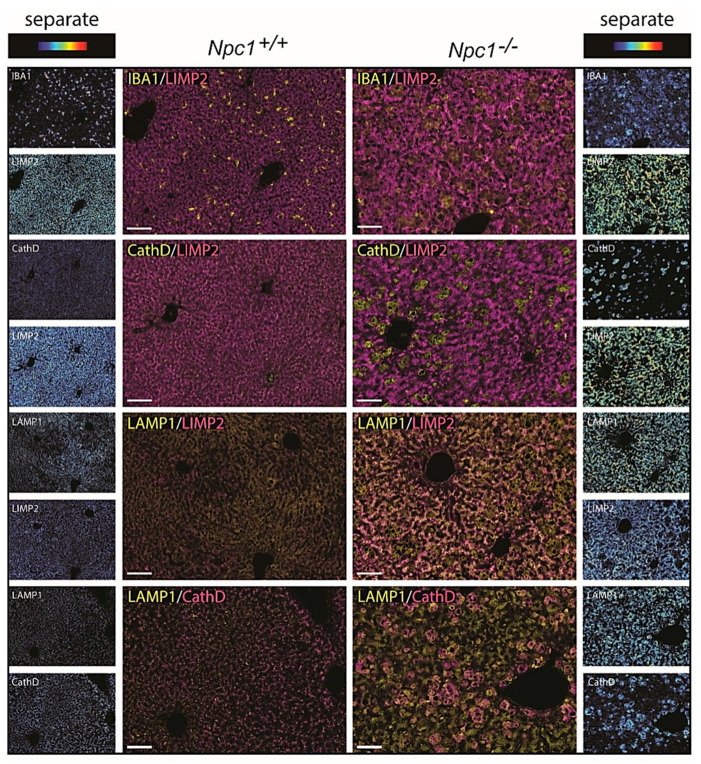
Immunohistochemistry NPC liver; “Composite” panels of immunostaining of *Npc1^+/+^* and *Npc1^−/−^* liver of 80-day-old mice. The first three panels show IBA1, cathepsin D, and LAMP1 in yellow and LIMP2 in magenta. The last panel shows LAMP1 in yellow and cathepsin D in magenta. Brightfield scans were analyzed using spectral imaging; separate images are displayed in heat-map intensity scale. Scale bar = 50 µm.

**Figure 6:**
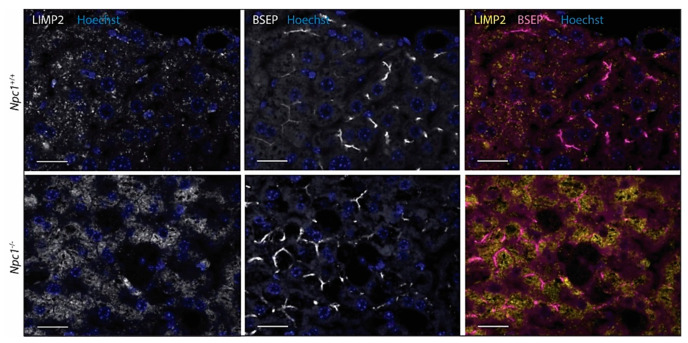
Immunofluorescence of LIMP2 and bile salt efflux pump (BSEP) in hepatocytes of *Npc1^+/+^* and *Npc1^−/−^* liver. Scale bar = 20 µm.

Contrary to matched control liver, the *Npc1*^−/−^ tissue showed the presence of IBA1-positive, lipid-laden macrophages (Figure 5 upper panel) [24]. These Kupffer cells were strongly stained for Cathepsin D (Figure 5 second panel). Likewise, GPNMB and galectin-3 staining was also abundant in these storage macrophages of the *Npc1*^−/−^ liver Supplementary Figure S3A. Unexpectedly, the analysis of LIMP2 revealed predominant labelling of hepatocytes in the *Npc1*^−/−^ liver (Figure 5). Increased expression of LAMP1 was present throughout the *Npc1^−/−^* liver, also in the macrophages that show limited staining for LIMP2 (Figure 5, third and last panels, as well as highlighted in Supplementary Figure S3B). Double-staining for LIMP2 and the bile salt efflux pump (BSEP) revealed preferential distribution of LIMP2 adjacent to the apical membrane in both *Npc1^+/+^* and *Npc1^−/−^* hepatocytes (Figure 6).

### 2.6. MiT/TFE Transcription Factors Regulating Lysosomal Biogenesis in NPC Liver

**Figure 7:**
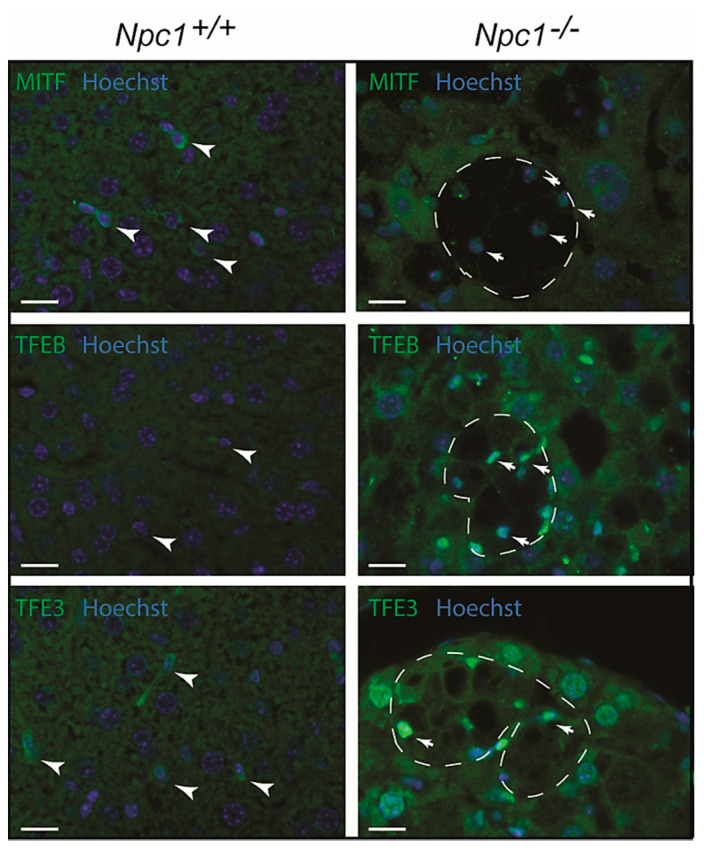
Immunofluorescence analysis of transcriptional regulators of lysosomal biogenesis in *Npc1^−/−^* liver of 80-day-old mice: MITF (top; melanocyte inducing transcription factor), TFEB (middle; transcription factor EB) and TFE3 (bottom; transcription factor E3); nucleus is depicted in blue (Hoechst); arrowheads indicate Kupffer cells in *Npc1^+/+^* mice; dashes outline clusters of Kupffer cells in livers of *Npc1^−/−^* mice; arrows indicate nuclear localization of analyzed transcription factors in *Npc1^−/−^* Kupffer cells; scale bar = 20 µm.

Cellular compensation for lysosomal dysfunction can occur through the initiation of a transcriptional program that drives biogenesis of lysosomes. This response has been extensively documented and is known to be driven by the microphthalmia-transcription factor E (MiT/TFE) subfamily of basic helix-loop-helix transcription factors (TFs), which include TFEB (transcription factor EB), TFE3 (transcription factor E3), and MITF (melanocyte inducing transcription factor) [30,31]. Immunohistochemical analysis of MITF, TFEB, and TFE3 was performed for *Npc1^−/−^* liver (Figure 7). Kupffer cells in the wild type liver contained cytoplasmic MITF (Figure 7). In the *Npc1^−/−^* condition, upregulation of MITF was observed with nuclear translocation in macrophages. Regarding TFEB, a similar trend was observed. However, in the case of TFE3, hepatocytes also showed nuclear enrichment of the transcription factor, in addition to the Kupffer cells.

Taken together, a nuclear localization of MiT/TFE transcription factors is observed in *Npc1^−/−^* liver of 80-day-old mice, especially in Kupffer cells (see Supplementary Figure S4). This may drive lysosome biogenesis and expression of autophagy genes. The remarkable upregulation of LIMP2 in hepatocytes correlates best with the nuclear localization of TFE3.

## 3. Discussion

For many decades, biochemical abnormalities in Niemann–Pick disease type C have been studied. Our present study focusses on abnormalities in GCase in the liver of mice that lack the lysosomal transmembrane protein NPC1 and consequently develop Niemann–Pick disease type C. Earlier investigations already showed that the livers of *Npc1*^−/−^ mice contain increased levels of GlcCer, the substrate of GCase, along with accumulation of cholesterol [7,24]. In parallel, GlcSph is increased in the *Npc1*^−/−^ liver. These studies indicate that local degradation of GlcCer by lysosomal GCase is impaired, which is followed by increased de-acylation through lysosomal acid ceramidase [8]. Moreover, livers of *Npc1*^−/−^ mice accumulate GlcChol, which points to an increased transglucosylation activity of GCase [9]. Earlier work also described accumulation of GPNMB positive foamy cells in the liver of *Npc1*^−/−^ mice, resembling Gaucher cells during a primary GCase deficiency (Gaucher disease) [24]. We examined GCase in more detail in livers of *Npc1*^−/−^ mice by making use of activity-based probes (ABPs) selectively labelling active enzyme molecules. Furthermore, by Western blotting, qPCR, and immunohistochemistry, we also examined other proteins known to be changed during GCase deficiency. ABP labelling confirmed reduction in active GCase content of livers of *Npc1*^−/−^ mice. Reduction in GCase was accompanied by a reciprocal increase of active GBA2 molecules in liver lysates of *Npc1*^−/−^ mice. Of note, in brains of *Npc1*^−/−^ mice, an increase in GBA2/GCase ratio was already observed previously [10] and genetic ablation or pharmacological inhibition of GBA2 in *Npc1*^−/−^ mice significantly increases life span [10]. The reduction of GCase following lysosomal cholesterol accumulation was recapitulated in vitro with cultured HEPG2 cells treated with a known NPC1 inhibitor, U18666A. Our immunohistochemical investigation of livers of *Npc1*^−/−^ mice confirmed the presence of characteristic storage cells that were positive for the macrophage marker IBA1. An increase in GPNMB in livers of 80-week-old *Npc1*^−/−^ mice was observed on RNA level, and by Western blot. The foamy macrophages were found to overexpress GPNMB, galectin-3, and cathepsin D.

In accordance to the earlier report by Peregrande et al., LIMP2 was found to be increased in the livers of *Npc1*^−/−^ mice. Unexpectedly, the immunohistochemical analysis of the *Npc1*^−/−^ livers revealed a very prominent expression of LIMP2 in hepatocytes and not in the lipid-laden macrophages. The pattern of LIMP2 staining differed from that of lysosome marker LAMP1 regarding subcellular localization. Interestingly, inducing lysosomal cholesterol accumulation in HEPG2 cells with U18666A caused no overexpression of LIMP2. These findings show that cultured cells do not provide a phenocopy of the polarized hepatocytes in the *Npc1*^−/−^ liver. The striking upregulation of LIMP2 in hepatocytes of the *Npc1*^−/−^ liver and apparent partial non-lysosomal localization is intriguing and warrants further discussion. As compared to Kupffer cells, *Npc1*^−/−^ hepatocytes demonstrate less prominent lysosomal storage [3]. It could be speculated that the upregulation of LIMP2 is instrumental to this. Heybrock et al. recently provided evidence that LIMP2 can assist efflux of cholesterol from lysosomes in an NPC1-independent manner. In NPC1-deficient cells, SREBP2 (sterol regulatory element binding transcription factor 2) -mediated transcription is upregulated in response to the reduced cholesterol level in the endoplasmic reticulum resulting from impaired sterol efflux from lysosomes. The transcription factor SREBP2 controls cholesterol homeostasis by stimulating transcription of gene-encoding proteins involved in the biosynthesis of cholesterol [32]. Generating a combined deficiency of NPC1 and LIMP2 was found by Heybrock et al. to increase SREBP2-mediated de novo synthesis of cholesterol, pointing to LIMP2-mediated cholesterol efflux from lysosomes during NPC1 deficiency [14]. Clearly, the efflux of cholesterol from lysosomes is primarily mediated by the NPC2/NPC1 pathway since individuals deficient in LIMP2 (KO mice and patients suffering from acute myoclonus renal failure syndrome, AMRF) show no signs of disturbed cholesterol metabolism [33]. If overexpression of LIMP2 serves as a compensatory mechanism to NPC1 deficiency, the question arises why such response does not seem to take place in the macrophage-like cells that transform into lipid storage macrophages. These cells rather seem to upregulate lysosomes in order to increase their storage capacity.

The apparent partial non-lysosomal location of LIMP2 in *Npc1*^−/−^ hepatocytes prompts the hypothesis that LIMP2 could be additionally involved in the export of cholesterol to the bile. In view of this hypothesis, examination of *Npc1*^−/−^ enterocytes may be of interest. It has been recognized for some time that cholesterol is exported from enterocytes into the intestinal lumen via so-called TICE (trans-intestinal cholesterol export) [34]. Possibly, LIMP2 could also be a player in this physiologically relevant process of cholesterol export.

In conclusion, our study on livers of *Npc1*^−/−^ mice has led to the discovery of a prominent upregulation of LIMP2 in hepatocytes in combination with a non-lysosomal location. This observation might point to a compensatory pathway to prevent cholesterol accumulation in hepatocytes. Our finding could give impetus to further studies on compensatory mechanisms in lysosomal storage disorders and the beneficial value of these processes. Specifically, the putative role of LIMP2 in cholesterol efflux to the bile in the context of NPC1 deficiency warrants further testing. Ideally, the flux of cholesterol into bile is measured by bile duct cannulation in *Npc^−/−^* mice crossbred with *Scarb2^−/−^* mice lacking LIMP2 [35,36].

## 4. Materials and Methods

### 4.1. Cell Culture Experiments

RAW264.7 cells and HEPG2 cells (TIB-71 and HB-8065 resp., American Type Culture Collection, Manassas, VA, USA) were cultured in DMEM (Dulbecco’s Modified Eagle Medium) containing 10% fetal calf serum, 1% glutamax and 0.2% antibiotics (penicillin-streptomycin; all purchased from Thermo Fisher Scientific, Eindhoven, The Netherlands) at 37 °C at 5% CO_2_. NPC1 inhibition was performed using 10 µM U18666 (U3633, Sigma-Aldrich, Zwijndrecht, The Netherlands) [9].

### 4.2. Animals

Mice heterozygous for a spontaneous truncation of the *Npc1* gene, BALB/c Nctr-Npc1m1N/J mice (#003092) were obtained from The Jackson Laboratory (Bar Harbor, Hancock County, ME, USA). Males and females of the two strains were crossed in-house to generate *Npc1*^−/−^, and wild type littermates (*Npc1*^+/+^). Mice received a normal chow diet and water ad libitum and were housed in a temperature- and humidity-controlled room with a 12-h light/dark cycle. National and local ethical committee approval was obtained for conducting animal experiments and laboratory animal welfare rules were enforced (DBC101698 and DBC17AC). In order to anesthetize mice, Hypnorm (0.315 mg/mL phenyl citrate and 10 mg/mL fluanisone) and Dormicum (5 mg/mL midazolam) was administered according to their weight (80 μL per 10 g bodyweight) and subsequent cervical dislocation was performed. Organs were dissected and fixed in 4% formalin for immunohistochemical analysis or snap-frozen for protein and mRNA analysis.

### 4.3. Electron Microscopy and Toluidine Blue Staining

For transmission electron microscopy, fresh liver was fixed in paraformaldehyde/glutaraldehyde (Karnovsky’s fixative) and post-fixed with 1% osmium tetroxide. The fixed tissue samples were block-stained with 1% uranyl acetate, dehydrated in dimethoxypropane, and embedded in epoxy resin LX-112. Furthermore, 1-µm-thick sections were stained with toluidine blue and imaged by brightfield microscopy (Leica DM5500B, Mannheim, Germany) with an HCX PL APO 63×/1.40−0.60 oil immersion objective. Ultrathin sections were stained with uranyl acetate, and lead citrate. Examination was performed using a Tecnai T12 transmission electron microscope (Thermo Fisher, Eindhoven, The Netherlands). Images were acquired using a digital transmission EM camera (Veleta digital camera, Münster, Germany, using Radius software from EMSIS Germany).

### 4.4. Immunohistochemistry and Immunofluorescence

Dissected tissue was fixed in 4% formalin (pH 7.0 by phosphate buffer) and embedded in paraffin. Embedded tissue was cut into 4 µm-thick sections, washed in 100% xylene to remove paraffin, and washed in 100% ethanol. Rehydration occurred through incubation in 96% ethanol, 70% ethanol, and miliQ water, respectively, and heat induced epitope retrieval (HIER) was performed at 98 °C for 10 min in 10 mM citric acid (pH 6). Tissues were washed and permeabilized in PBS/0.01% Tween-20 (CAS 9005-64-5, Sigma, Zwijndrecht, The Netherlands) and incubated with primary antibodies goat-anti-GPNMB (AF2330; R&D Systems, Abingdon, UK), rat-anti-galectin-3 clone M3/38 (MABT51, Millipore; pre-blocked with 5% mouse serum and centrifuged), rabbit-anti-cathepsin D (antiserum was prepared in our laboratory), rabbit-anti-LIMP2 (NB400-129, Novus Biologicals, Abingdon, UK), rat-anti-BSEP (PA5-78690, Thermo Fisher), rabbit-anti-MITF (AB122982, Abcam, Cambridge, UK), rabbit-anti-TFEB (A303-673A, Bethyl Lab Inc., Leiden, Netherlands), or rabbit-anti-TFE3 (HPA023881, Sigma). For Supplementary Figure S3B,C, goat-anti-LIMP2 was used (SAB2501242, Sigma). Antibodies were diluted in PBS/5% antibody diluent (ScyTek Laboratories, Duiven, The Netherlands). In order to prepare immunohistochemistry for spectral imaging, slides were washed, incubated with poly-AP(alkaline phosphatase)-conjugated donkey anti-goat IgG (A16008, Life Technologies, Eindhoven, The Netherlands), goat-anti-rat IgG (Brightvision, ImmunoLogic, Klinipath, Duiven, The Netherlands), or goat-anti-rabbit IgG (BrightVision) to their respective primary antibody. Bound AP was visualized through incubation with AP substrate VectorBlue (SK-5300; Vector Laboratories, Burlingame, CA, USA) in presence of 0.2 mM levamisole to inhibit endogenous alkaline phosphatase activity. For double staining, slides were washed and subjected to a second HIER to inactivate and remove existing antibody-antigen bonds while leaving the precipitated chromogen unchanged [37]. Sections were incubated with goat-anti-GPNMB (AF2330), rat-anti-galectin-3 clone M3/38 (MABT51), rabbit-anti-SCARB2 (NB400-129) or rabbit-anti-ZKSCAN3 (20800-1-AP, Proteintech, Uden, The Netherlands), washed, and incubated with poly-AP goat anti-goat or rat IgG. Development of signal was performed through incubation with VectorRed AP substrate (SK-5100; Vector Laboratories) in presence of 0.2 mM levamisole. Sections were mounted with VectaMount (Vector Laboratories). Images were obtained by brightfield microscopy (Leica DM5500B) with an HC PLAN APO 20×/0.70 objective. Nuance imaging system (Perkin Elmer, Hopkinton, MA, USA) allowed acquisition of multispectral data sets from 420 to 720 nm with 10 nm intervals. Single-stained sections were used to define the spectral properties of each color in order to unmix the double staining patterns. Construction of composite images was done by Nuance 3.0.2 software, which rendered display intensity heat maps for single channels and subsequently allowing a color universal design. With respect to immunofluorescence analysis, aforementioned primary antibodies were visualized by secondary donkey antibodies against mouse, rabbit, or goat IgG, conjugated with Alexa FluorTM 647 (A31571, A31573, and A21447 resp., Molecular Probes, Eindhoven, The Netherlands) or Alexa FluorTM 488 (A21202, A21206, and A11055 resp., Molecular Probes). For detection of galectin-3, goat-anti-rat IgG conjugated to TxR was used (3052-07, Southern Biotech, Uithoorn, The Netherlands). Images were obtained by fluorescence microscopy (Leica DM5500B) with an HCX PL APO 63×/1.40−0.60 oil objective.

### 4.5. Enzyme Activity Assays

Protein quantification of cell and tissue homogenates was assessed by bicinchoninic acid assay (Thermo Fisher Scientific, 23225). Equal protein amounts were used for enzyme activity assays. GCase activity was assayed using 4-methylumbelliferyl (4-MU) substrate beta-D-glucopyranoside (44059, Glycosynth, Warrington, UK) in McIlvaine buffer, pH 5.2, with 0.1% (*w/v)* BSA [38].

### 4.6. Activity-Based Probe Analysis

Where stated, homogenates of tissue and cells were labelled with excess of activity-based probe (ABP) conjugated to a fluorescent dye as earlier described [39]. When GCase was labelled in lysates of cultured cells (Figure 4), ultrasensitive labeling of all active GCase molecules was performed using 100 nM ABP-ME569 (Cy5) [39]. Incubation was performed at 100 nM for 1 h (0.5−1% DMSO (dimethylsulfoxide)) on ice for homogenates, for living cells at 37 °C. In homogenates of tissue (Figure 2), GCase and GBA2 were labelled using a broad specificity ABP for retaining β-exoglucosidases, ABP-JJB367 (containing Cy5) [40]. Labelling occurred at 200 nM ABP-JJB367 at pH 5.8 (0.5−1% DMSO) for 1 h on ice. Samples were denatured and separated by SDS-PAGE. Detection of fluorescence in wet gel slabs was performed using a Typhoon FLA 9500 fluorescence scanner (GE Healthcare, Eindhoven, NB, The Netherlands). Far red fluorescence (ME569 and JJB367) was detected using λEX 635 nm and λEM ≥ 665 nm. After imaging, gels were either stained by Coomassie G250 for total protein and scanned on ChemiDoc MP imager (Bio-Rad, Veenendaal, The Netherlands) or used for Western blotting.

### 4.7. Western Blot Analysis

Frozen tissue samples and cultured cells were lysed in KPi lysis buffer (25 mM K2HPO4/KH2PO4, pH 6.5, 0.1% (*v:v*) Triton X-100) supplemented with protease inhibitors (Roche) and sonicated 5× for 1 s with 9 min intervals (amplitude 25%). Equal quantities of protein as assessed by bicinchoninic acid assay (Thermo Fisher Scientific, 23225) were resuspended in Laemmli buffer and denatured at 95 °C. Proteins were subsequently separated by SDS-PAGE using a 10% acrylamide gel and transferred to 0.2 µm nitrocellulose membrane (#1704159, Bio-Rad). Blocking of membranes occurred in 5% (*w:v*) bovine serum albumin (Sigma, A1906) solution in PBS/0.1% Tween-20 (Sigma, P1379) for 1 h at room temperature (RT). Primary antibodies used were targeted against GPNMB (AF2330; R&D Systems), LIMP2 (NB400-129, Novus Biologicals), and galectin-3 (MABT51, Millipore). Furthermore, rabbit-anti-LAMP1 was used (ab24170, Abcam) and mouse-anti-tubulin (Cedarlane, CLT 9002, Burlington, ON, Canada) was used as loading control. Proteins were detected by using specific secondary conjugated antibodies (Alexa FluorTM 488/647) (Molecular Probes). Detection of immunoblots was performed using a Typhoon FLA 9500 fluorescence scanner (GE Healthcare).

### 4.8. RNA Extraction and Real-Time PCR

Total RNA from liver or cell culture was extracted by means of the NucleoSpin II extraction kit (Macherey Nagel, Leiden, The Netherlands) according to manufacturer’s protocol. RNA concentrations were measured (DeNovix DS-1) and equal amounts of RNA were used for cDNA synthesis according to the manufacturer’s protocol (Invitrogen). Real time qPCR was performed using Bio-Rad CFX96 Touch real-time PCR detection system (Bio-Rad Laboratories). The following genes were analyzed: *Gpnmb* (forward primer (FW): caggaatgatttgggactgacc; reverse primer (RV): ccgggaacctgagatgctg), *Lgals3* (FW: tacaggtgcctgctcacttg; RV: ggacctgacctgtctcagga), *Lamp1* (FW: acactgctgacgtgtcttgc; RV: gtgagctgatgcccagtgta), *Atp6v1a* (FW: ctacccaaaatccgcgatgag, RV: ccatgtcaccttccaatcgaa), *Asah1* (FW: tgcccagacccttgtatagg, RV: aagaggccttgagccttagc), *Ctsd* (FW: ctgagtggcttcatgggaat, RV: cctgacagtggagaaggagc), *Tnfa* (FW: acggcatggatctcaaagac, RV: agatagcaaatcggctgacg), *Ccl2* (FW: aggtccctgtcatgcttctgg, RV: ctgctgctggtgatcctcttg), *Iba1* (FW: ccgaggagacgttcagctac, RV: gatctcttgcccagcatcat). Acidic ribosomal phosphoprotein 36B4 (P0, FW: ggacccgagaagacctcctt, RV: gcacatcactcagaatttcaatgg) was used as reference gene.

### 4.9. Lipid Measurements

Glycosylated lipids (GlyCer, GlySph, and GlyChol) in livers were measured by LS-MS/MS (liquid chromatography with tandem mass spectrometry) procedures using 13C isotope encoded standards as described earlier [7,9,24].

## Supplementary Materials

The following are available online at https://www.mdpi.com/1422-0067/22/5/2532/s1, Figure S1: Quantification of GCase (left) and GBA2 (right) band intensities shown in Figure 2C, Figure S2: Quantification of LIMP2 band intensities on immunostained western blot as shown in Figure 4A, Figure S3: Immunohistochemistry NPC liver, Figure S4: Immunohistochemical analysis showing ‘composite panels’ of MiT/TFE family members and IBA1 in *Npc1^+/+^*- and *Npc1^-/-^*-liver of 80-days-old mice.

## Abbreviations

ABP	activity based probe
AMRF	acute myoclonus renal failure syndrome
CCL2	C−C motif chemokine ligand 2
CCL18	C−C motif chemokine ligand 18
GBA2	glucosylceramidase beta 2
GCase	glucosylceramide beta
GD	Gaucher disease
GlcCer	glucosylceramide
GlcChol	glucosylated cholesterol
GlcSph	glucosylsphingosine
GPNMB	glycoprotein non metastatic protein B
HMG-CoA reductase	3-hydroxy-3-methylglutaryl-CoA reductase
IBA1	allograft inflammatory factor 1
LAMP1	lysosomal associated membrane protein 1
LIMP2	sterol regulatory element binding transcription factor 2
MiT/TFE	microphthalmia-transcription factor E
MITF	melanocyte inducing transcription factor
NPC	Niemann–Pick type C
NPC1	NPC intracellular cholesterol transporter 1
NPC2	NPC intracellular cholesterol transporter 2
SREBP2	scavenger receptor class B member 2
TFE3	transcription factor E3
TEM	transmission electron microscopy
TFEB	transcription factor EB
TNFα	tumor necrosis factor alpha
ZKSCAN3	zinc finger with KRAB and SCAN domains 3
