# Supplementary material for: GCase and LIMP2 Abnormalities in the Liver of Niemann Pick Type C Mice
Source: Int J Mol Sci. 2021 Mar 3;22(5):2532. doi: 10.3390/ijms22052532 (PMC7959463; doi:10.3390/ijms22052532)
Supplement: Supplementary file 1 [file ijms-22-02532-s001.pdf]

Supplementary figures

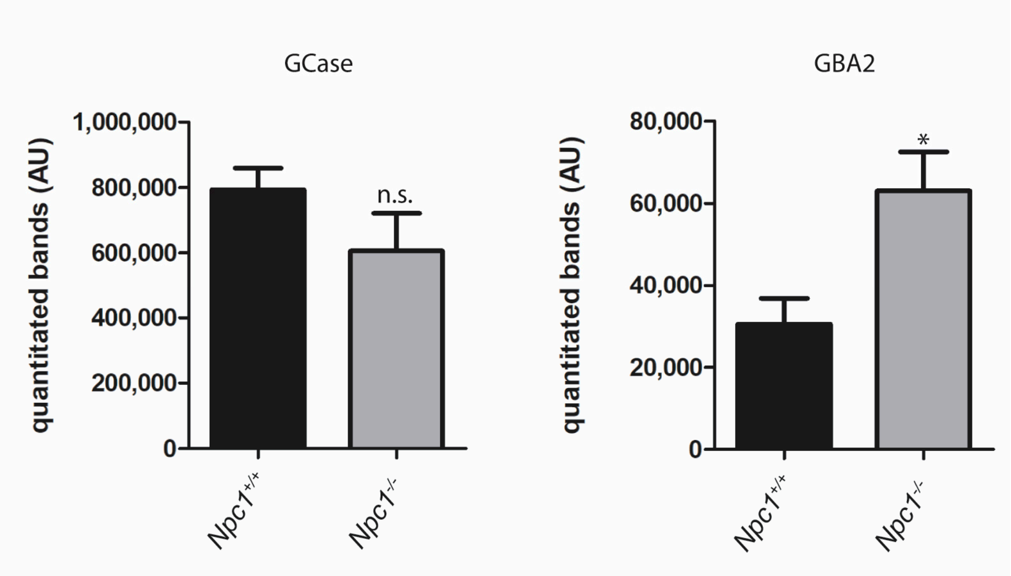

Figure S1. Quantification of GCase (left) and GBA2 (right) band intensities shown in figure 2C; GCase and GBA2 specific ABP was employed to fluorescently label the enzymes and subsequently visualized by fluorescence scanning after SDS-PAGE total active GCase and GBA2.

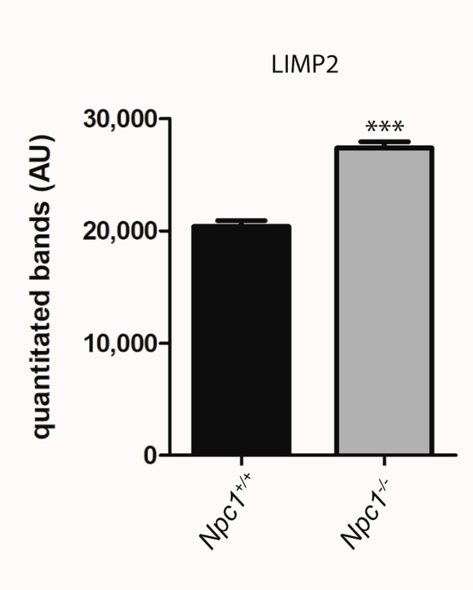

Figure S2. Quantification of LIMP2 band intensities on immunostained western blot as shown in figure 4A.

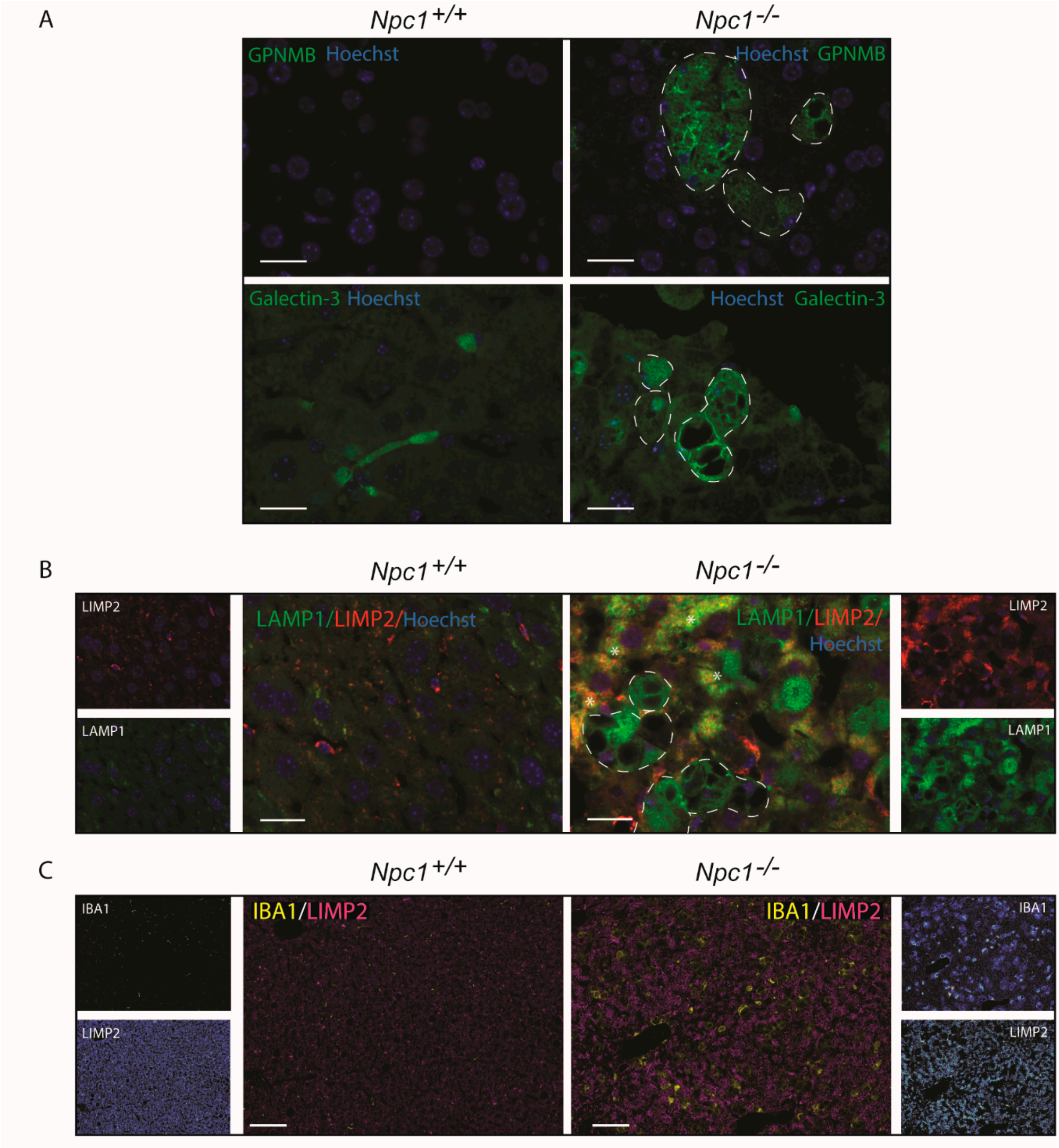

Figure S3. Immunofluorescence of $Npc^{+/+}$ and $Npc^{-/-}$ liver (**A**) Immunofluorescence microscopy of GPNMB and galectin-3. Scale bar = 20 µm; dashes outline clusters of Kupffer cells in livers of Npc1-/- mice; scale bar=20µm. (**B**) Immunofluorescence microscopy of LIMP2 and LAMP1. Scale bar = 20 µm; dotted line indicates the borders of the $Npc1^{-/-}$ deficient Kupffer cell; (**C**) Verification of LIMP2 pattern by alternative antibody; composite' panels of immunostaining of $Npc1^{+/+}$- and $Npc1^{-/-}$-liver of 80-days-old mice: IBA1 is depicted in yellow and LIMP2 in magenta. Brightfield scans were analyzed using spectral imaging; separate images are displayed in heat-map intensity scale. Scale bar = 50 µm.

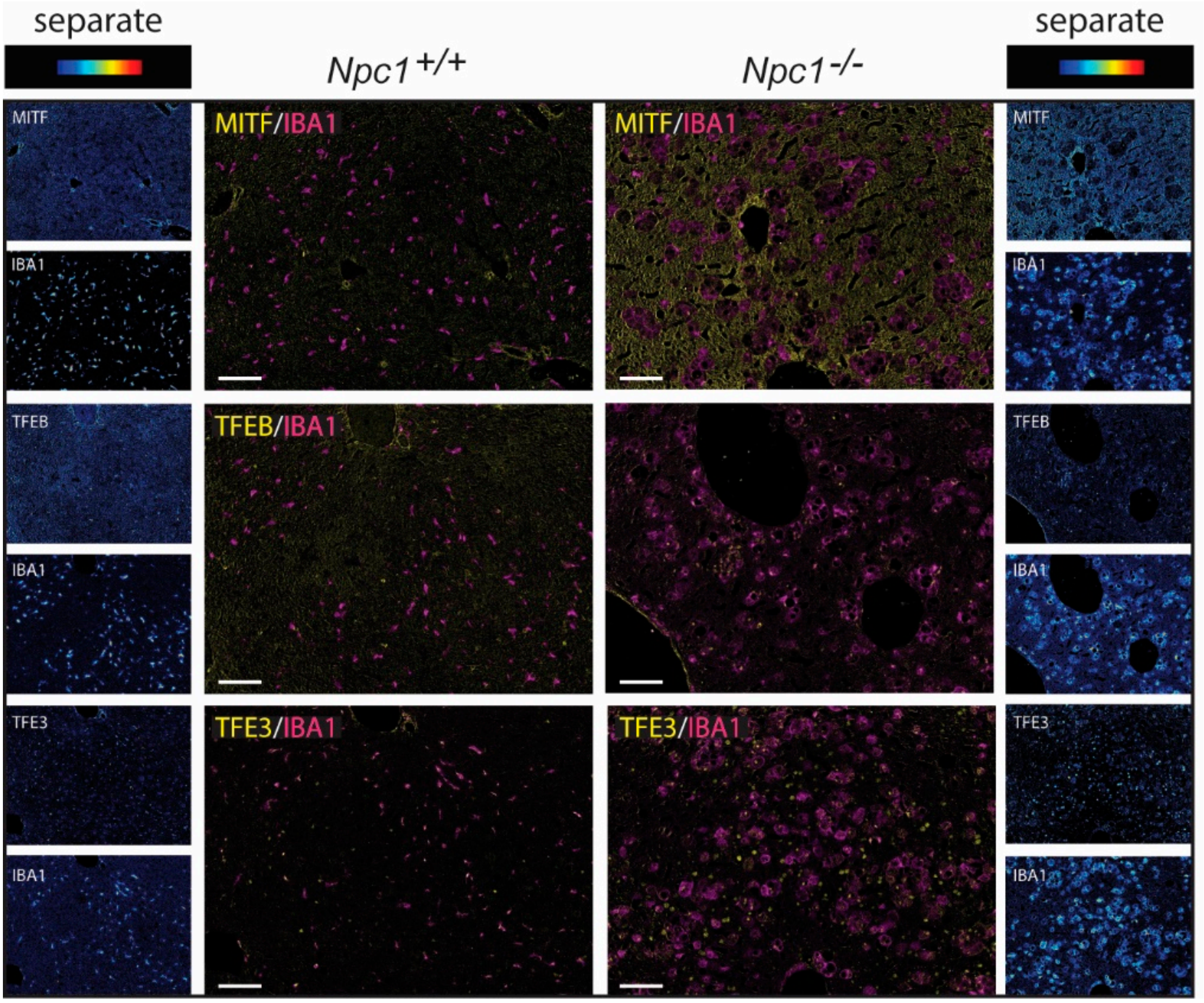

Figure S4. Immunohistochemical analysis showing 'composite panels' of MiT/TFE family members and IBA1 in Npc1$^{+/+}$- and Npc1$^{-/-}$-liver of 80-days-old mice; MITF, TFEB and TFE3 are depicted in yellow and IBA1 in magenta. Brightfield scans were analyzed using spectral imaging; separate images are displayed in heat-map intensity scale. Scale bar = 50 μm.
